# Investigation of different nitrogen reduction routes and their key microbial players in wood chip-driven denitrification beds

**DOI:** 10.1038/s41598-017-17312-2

**Published:** 2017-12-05

**Authors:** Victoria Grießmeier, Andreas Bremges, Alice C. McHardy, Johannes Gescher

**Affiliations:** 1 Department of Applied Biology, Institute for Applied Biosciences, Karlsruhe Institute of Technology (KIT), Karlsruhe, Germany; 2 Computational Biology of Infection Research, Helmholtz Centre for Infection Research, Braunschweig, Germany; 3 German Center for Infection Research (DZIF), partner site Hannover-Braunschweig, Braunschweig, Germany; 4 Institute for Biological Interfaces, Karlsruhe Institute of Technology (KIT), Eggenstein-Leopoldshafen, Germany

## Abstract

Field denitrification beds containing polymeric plant material are increasingly used to eliminate nitrate from agricultural drainage water. They mirror a number of anoxic ecosystems. However, knowledge of the microbial composition, the interaction of microbial species, and the carbon degradation processes within these denitrification systems is sparse. This study revealed several new aspects of the carbon and nitrogen cycle, and these findings can be correlated with the dynamics of the microbial community composition and the activity of key species. Members of the order Pseudomonadales seem to be important players in denitrification at low nitrate concentrations, while a switch to higher nitrate concentrations seems to select for members of the orders Rhodocyclales and Rhizobiales. We observed that high nitrate loading rates lead to an unpredictable transition of the community’s activity from denitrification to dissimilatory reduction of nitrate to ammonium (DNRA). This transition is mirrored by an increase in transcripts of the nitrite reductase gene *nrfAH* and the increase correlates with the activity of members of the order Ignavibacteriales. Denitrification reactors sustained the development of an archaeal community consisting of members of the Bathyarchaeota and methanogens belonging to the Euryarchaeota. Unexpectedly, the activity of the methanogens positively correlated with the nitrate loading rates.

## Introduction

Since the onset of industrialization in the 19^th^ century, innovations in the agricultural sector were required to feed a continuously growing global population^1,2^. Fertilizers were a key factor in the quest to overcome food shortages and their use increased steadily beginning in the 1950s and 1960s^3,4^. This led to an increase in nitrogen in the environment^5^. As a result, the natural balance between terrestrial nitrogen inputs via nitrogen fixation and the return of nitrogen to the atmosphere via denitrification became disturbed in favor of nitrogen inflow^2^. This can cause the pollution of sensitive ecosystems, especially ground and surface waters in close proximity to agricultural environments. A consequence of ground water pollution has been observed in Germany where around 35% of the 723 measuring points in 2010 were heavily polluted by 10–50 mg L^−1^ nitrate. Of these, 14% exceeded even the threshold value for nitrate in drinking water (50 mg L^−1^)^6^. Moreover, the European Commission referred Germany to the EU court because Germany was not able to reduce the fertilizer and manure inputs^7^. Germany is no exemption from the general trend as agricultural drainage water leads to a nitrate contamination of ground and surface waters at least in many industrial countries^8–12^. All this leads to extensive eutrophication of aquatic systems, which can cause harmful algae blooms, hypoxia, and even anoxia^1^. Since land shortages are constantly increasing, the challenges associated with minimizing the impact of agriculture on closely related preserved areas will continue to increase as well.

One approach for reducing nitrate in ecosystems is the use of denitrification beds, which provide a decentralized wastewater treatment. Inexpensive and commonly available carbon sources added to these beds can sustain microbial denitrification. Under anoxic conditions, NO_3_^−^ replaces oxygen as an alternative electron acceptor and is reduced via different intermediates (NO_2_^−^, NO, N_2_O) to N_2_, which is released to the atmosphere. Unfortunately, there is also the possibility that the denitrification process in these beds is incomplete and the greenhouse gas N_2_O (300 times more potent that CO_2_)^13^ is the end-product. Moreover, a potential risk of methane production in denitrification beds exists if nitrate concentrations are low^14^. The greenhouse gas methane is around 30-fold (including indirect effects) more potent than carbon dioxide^13^. An elimination of nitrogen from waste or drainage waters could also be achieved via anaerobic ammonium oxidation (Anammox), a process that is catalyzed by members of the Planctomycetales. Here, ammonium serves as electron donor and nitrite as electron acceptor. Dinitrogen is formed as the end product. This process could lead to an efficient N-removal especially in field denitrification beds that are in place to treat waters with high ammonium concentrations^15^. A competing microbial nitrate reduction process that could occur in denitrification systems is the dissimilatory nitrate reduction to ammonium (DNRA). An important factor for the incidence of either denitrification or DNRA seems to be the availability of electron donor (degradable carbon) and acceptor (nitrate), as it was shown, that nitrate is reduced to ammonium under anoxic and electron donor-rich as well as nitrate limiting (high C_org_/NO_3_^−^ ratio) conditions. In contrary, denitrification seems to dominate in environments with excess of nitrate^16,17^. Therefore, systems that do not favor DNRA will most effectively prevent eutrophication.

The general requirements of such denitrification plants include low construction costs, durability^5^, and self-sufficiency. A study conducted by Robertson and Cherry (1995) showed that wood chips serve as a suitable and slowly degrading carbon source for denitrifying microorganisms^18,19^. Previous research quantified the amount of denitrifying bacteria via quantitative PCR (qPCR) targeting key denitrification genes^20,21^. Using a metagenomic approach, another study analyzed the bacterial diversity and key enzymes involved in the hydrolysis of poplar wood chips under anoxic conditions without the influence of nitrate^22^. Here, especially members of the Clostridiales and Bacteroidetes were identified as organisms involved in the hydrolysis of cellulose^22^. However, less is known about the exact microbial composition and metabolic processes occurring in denitrification beds. This is of special importance because it is currently unknown (I) if similar rules for process conditions that suppress DNRA as stated above also apply for denitrification beds, (II) how the hydrolysis of lignocellulose is connected to the denitrification process and (III) if methanogenesis could be a competing process for the fate of carbon and electrons in the denitrification beds.

This study was motivated by a consideration of the basic requirements of an environmental denitrification system and by questions regarding the microbiome composition, the cooperative activity of such a system and its reaction towards different nitrate loading rates. Therefore, we 1) developed laboratory denitrification reactors using wood chips as a carbon source to investigate denitrification potential and the development of side-products (such as nitrite, ammonium, and methane), especially when the nitrate loads increased, and 2) analyzed the microbial community structure and activity within these reactors. A fen called *Mürmes* in the Vulkaneifel (Germany), that is surrounded and influenced by drainage water from agricultural areas, was chosen as a field site^23^.

## Results

### Nitrate elimination efficiency

**Figure 1:**
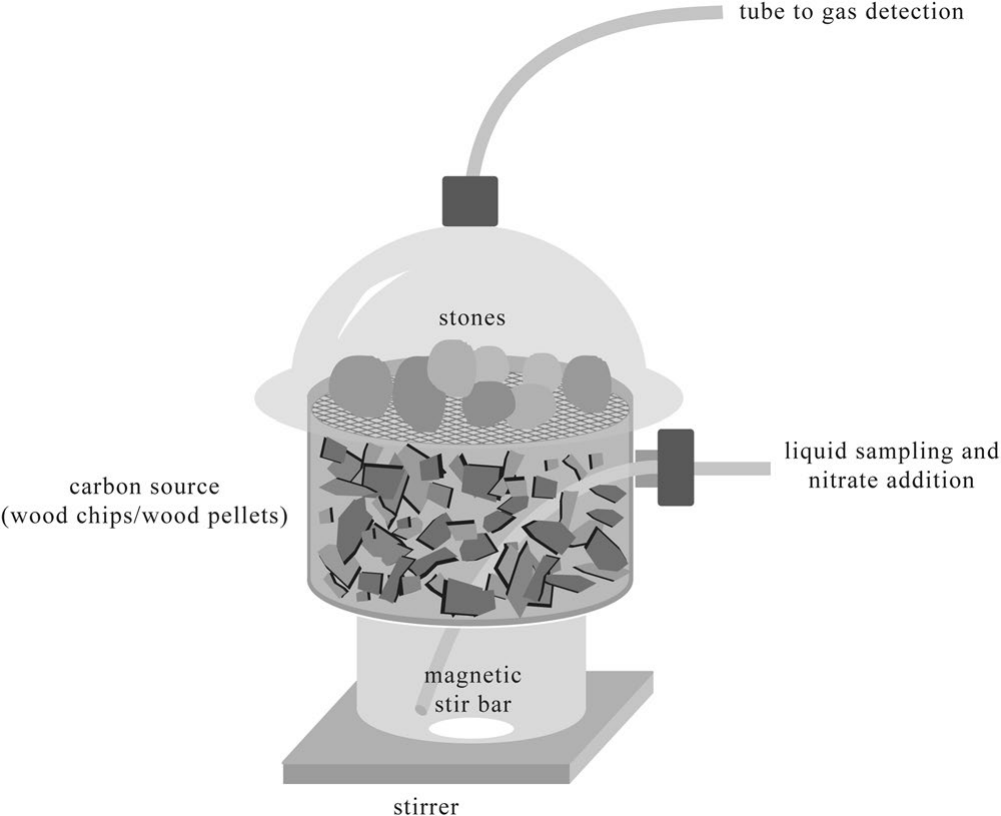
Laboratory setup. Depiction of the laboratory reactor filled with wood chips as a carbon source.

**Figure 2:**
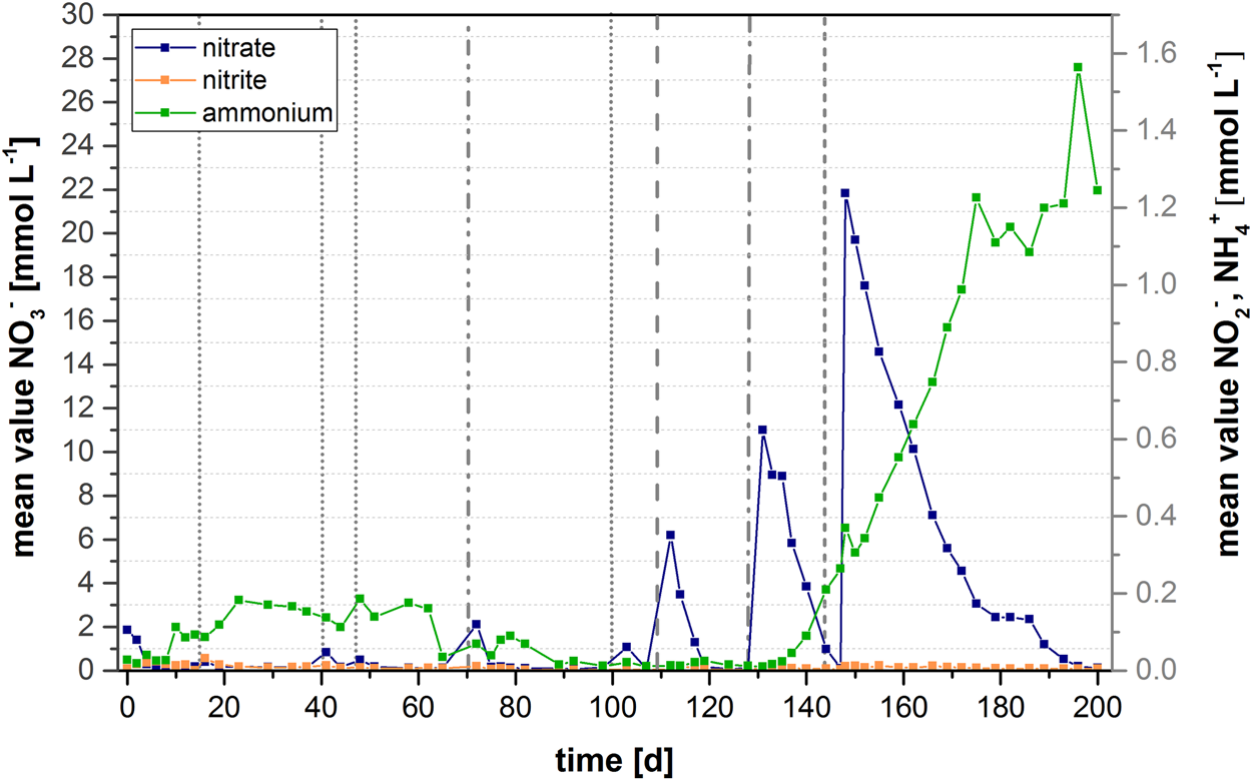
Development of nitrogen species in the laboratory reactor. Mean values for nitrate, nitrite, and ammonium in the wood chip reactors during the study. Dashed vertical lines indicate different amounts of nitrate added:  = 1×(1.18 mmol L^−1^) nitrate addition on day 15/41/48/103,  = 2 × (2.36 mmol L^−1^) nitrate addition on day 72,  = 5 × (5.9 mmol L^−1^) nitrate addition on day 112,  = 10 × (11.8 mmol L^−1^) nitrate addition on day 131, and  = 20 × (23.6 mmol L^−1^) nitrate addition on day 148.

This study aimed to establish the limits of nitrate elimination by a natural community and to analyze the community composition and activity under different nitrate loading rates. Our hypothesis was that the nitrate loading rate could have an influence on the physiology and composition of the microbiome. Laboratory reactors (Fig. 1) were inoculated with water samples from nitrate-contaminated drainages running into the *Mürmes* fen. Wood chips were chosen as carbon and electron source because of the promising results of earlier studies^5,14,24^. Figure 2 illustrates the average concentrations of the various nitrogen compounds found in the laboratory reactors during this study. A detailed analysis of each triplicate is depicted in the supplemental material (Fig. S1).

A rapid nitrate reduction was observed immediately following the addition of NO_3_^−^ (Fig. 2). Nitrate was almost completely reduced after each addition. Moreover, no NO_2_^−^ accumulation was detected during the experiment. After day 72, the behavior of the reactors with respect to higher nitrate loading rates was analyzed. Interestingly, the NO_3_^−^ elimination rate in the reactors decreased after the addition of nitrate at a concentration ten times higher than that observed in the field (on day 131). While the nitrate concentration decreased with a average rate of 1.3 mmol per day after the additon of 5.9 mmol L^−1^ this rate slowed down by 36% after the addition of 11.8 mmol L^−1^ NO_3_^−^. Moreover, the addition of the latter concentration altered the NH_4_^+^ concentration in the wood chip reactors, and a continuous increase with an average rate 0.06 mmol per day was observed until the end of the experiment. The increase in ammonium occurred also in a subsequent experimental replicate, which confirms that DNRA plays a role after adding high nitrate concentrations to the reactors (data not shown).

### Carbon compounds

**Figure 3:**
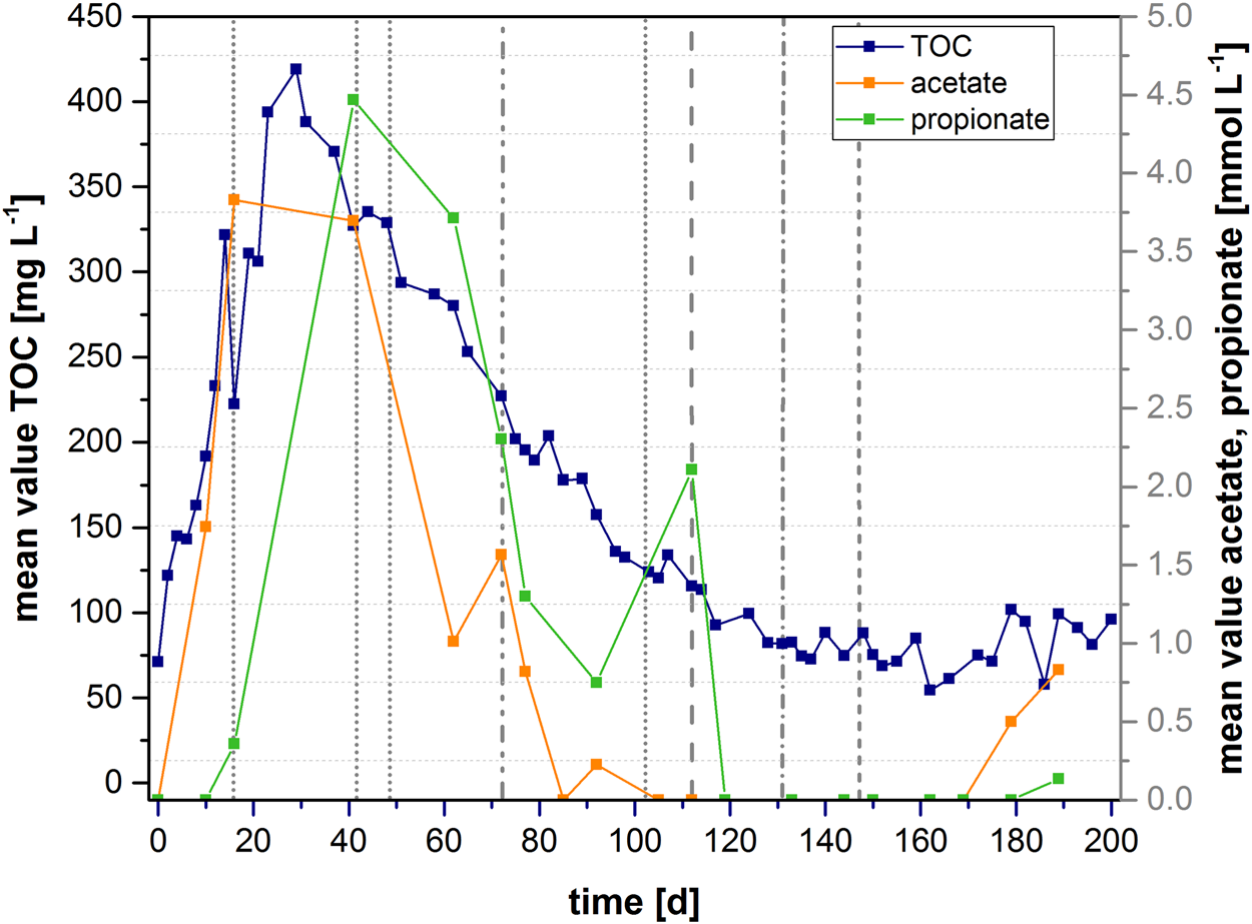
Development of carbon compounds in the laboratory reactor. Mean values of TOC, acetate, and propionate in the wood chip reactors during the study. Dashed vertical lines indicate different amounts of nitrate added:  = 1 × (1.18 mmol L^−1^) nitrate addition on day 15/41/48/103,  = 2 × (2.36 mmol L^−1^) nitrate addition on day 72,  = 5 × (5.9 mmol L^−1^) nitrate addition on day 112,  = 10 × (11.8 mmol L^−1^) nitrate addition on day 131, and  = 20 × (23.6 mmol L^−1^) nitrate addition on day 148.

The suitability of a carbon source for placement in denitrification beds is characterized by (1) its cost, (2) its durability, (3) the release rate of soluble carbon sources (which could lead to a eutrophication of the preserved area), and (4) its potential for forming climate gases during microbial degradation. Figure 3 shows the average concentrations of total organic carbon (TOC) and the concentrations of the detectable carboxylic acids (acetate and propionate) in the reactors during the study period. A detailed analysis of each triplicate experiment is depicted in the supplemental material (Fig. S2).

The TOC concentration increased during the first several days and reached a peak on day 29, after which it decreased until it reached a stable level of roughly 100 mg L^−1^ TOC. This concentration was observed from day 100 until the end of the experiment. The reactors contained detectable amounts of propionate and acetate until day 112 of the experiment. The concentrations of both compounds decreased, possibly as a result of their consumption by denitrifying microorganisms. Both carboxylic acids were undetectable during the addition of nitrate at a concentration of 11.8 mM L^−1^. On day 170, the acetate concentration started to increase again in two of the three reactors.

### Gas production

**Figure 4:**
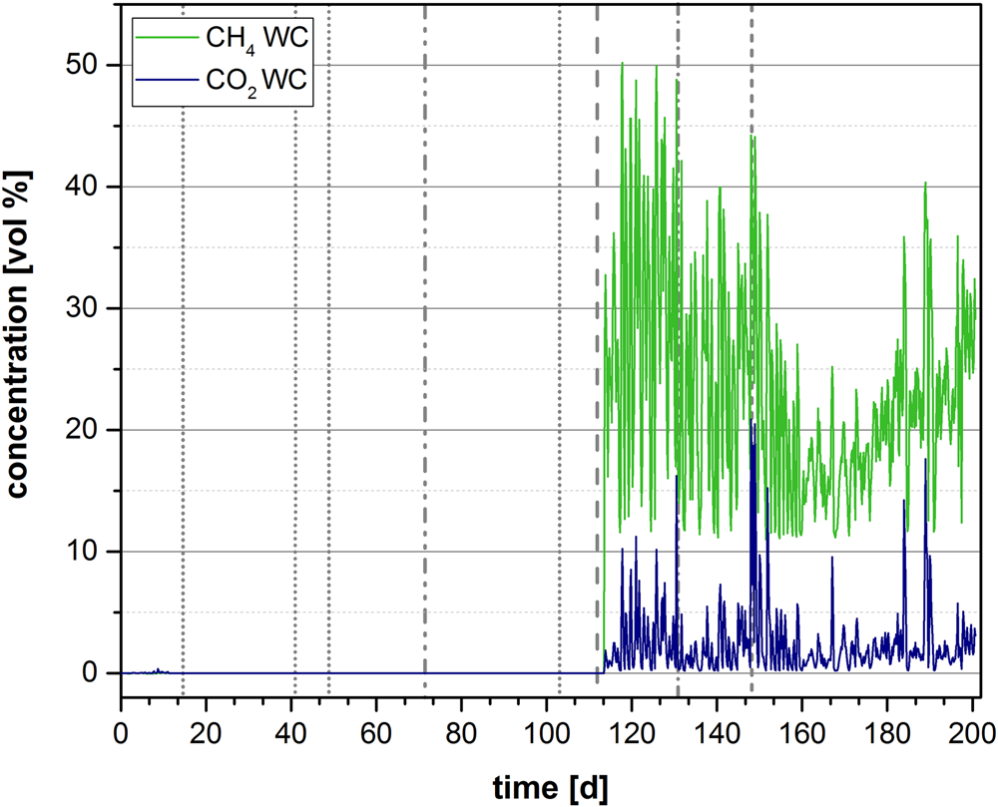
Gas production in the laboratory reactor. Development of emerging gases (CH_4_, CO_2_) in percent by volume during the experiment. WC: wood chip triplicate. Dashed vertical lines indicate different amounts of nitrate added:  = 1 × (1.18 mmol L^−1^) nitrate addition on day 15/41/48/103,  = 2 × (2.36 mmol L^−1^) nitrate addition on day 72,  = 5 × (5.9 mmol L^−1^) nitrate addition on day 112,  = 10 × (11.8 mmol L^−1^) nitrate addition on day 131, and  = 20 × (23.6 mmol L^−1^) nitrate addition on day 148.

Figure 4 shows CO_2_ and CH_4_ concentrations over the course of the experiment. The wood chip reactors began to produce CH_4_ and CO_2_ after day 112 when 5.9 mmol L^−1^ of NO_3_^−^ was added to the reactors. Prior to that time point, the gas counter did not detect any gas production. After day 112, a continuous cycle of increasing and decreasing gas levels of roughly 6–8 hours was observed. Similar cycles were detected for CO_2_, but at a lower concentration than CH_4_.

### Microbial community composition

Studies of the key players involved in nitrate elimination and cellulose hydrolysis in denitrification beds is lacking. To determine which Bacteria and Archaea might be involved in the nitrate reduction process, amplicon sequencing of the 16S rRNA genes and a metatranscriptomic analysis were conducted for low (time point A) and high (time point B) nitrate conditions, respectively. These two time points were chosen, because increasing ammonium concentrations led to the assumption that there was a switch in the microbial activity, which potentially could correlate with a change in the microbial composition of the biocenosis. The relative abundance of bacterial and archaeal operational taxonomic units (OTUs) in the inoculum, biofilm, and planktonic phase of the reactors was analyzed. Biofilm and planktonic samples were analyzed separately since the two areas of growth could offer completely different conditions regarding for instance the substrate availability or the local pH. Moreover, a differential abundance analysis was conducted to reveal the statistical relevance of the observed community changes between time point A and B.

The community composition in the wood chip reactors varied when low (time point A) and high (time point B) nitrate conditions and the sessile and planktonic phases were compared. As expected, the inoculum differed in the microbial composition compared to the reactors, but a few bacterial orders from the inoculum remained in the wood chip reactors. For example, members of the order Rhodocyclales could be found in every sample, while Planctomycetales and Rhizobiales were detectable in all samples except for the planktonic phase under low nitrate conditions (time point A). Members of the Rhodocyclales and Rhizobiales were more abundant at time point B (see Supplementary Table S5).

Pseudomonadales, Xanthomonadales, and Verrucomicrobiales only dominated in the solid phase of the reactors under low NO_3_^−^ conditions (time point A), while members of the order Burkholderiales were detectable in the inoculum and preferentialy in samples obtained under high nitrate concentrations (time point B) (Table S5).

Organisms belonging to the orders Spirochaetales, Bacteroidales, Sphingobacteriales, and Anaerolinales were only detected in the laboratory reactors, regardless of whether the NO_3_^−^ concentration was high or low. But the differential abundance analysis showed a higher presence for members of Anaerolineales for time point B and for Bacteroidales for time point A (Table S5).

Interestingly, members of the order Ignavibacteriales were detectable only in the reactors after addition of high concentrations of NO_3_^−^. Differential abundance testing confirmed a higher occurrence of all members of the Ignavibacteriales for time point B (see Table S5). Of note, Clostridia were detectable only under low nitrate concentrations (Table S5). They seemed to be replaced by other fermentative organisms belonging to the Anaerolineales under high nitrate conditions.

Archaeal sequences were also detected, and the majority of these sequences were related to uncultured members of the phylum Bathyarchaeota, formerly known as the Miscellaneous Crenarcheota Group (MCG). Bathyarchaeota have been suggested to play a major role in the methane cycle because members of this phylum were identified to be methylotrophic methanogens^25^. Moreover, Lazar *et al*. found in subgroups of the Bathyarchaeota genes, which seem to be involved in the reduction of nitrite to ammonium (DNRA) (such as *nrfD*, *nirB* and *nirD* ^26^), suggesting that these organisms could also play a role in the nitrogen cycle. Aside from Bathyarchaeota, methanogenic archaea belonging to the Euryarchaeota were detected. The occurrence of methanogens correlates well with CH_4_ production in these reactors.

**Figure 5:**
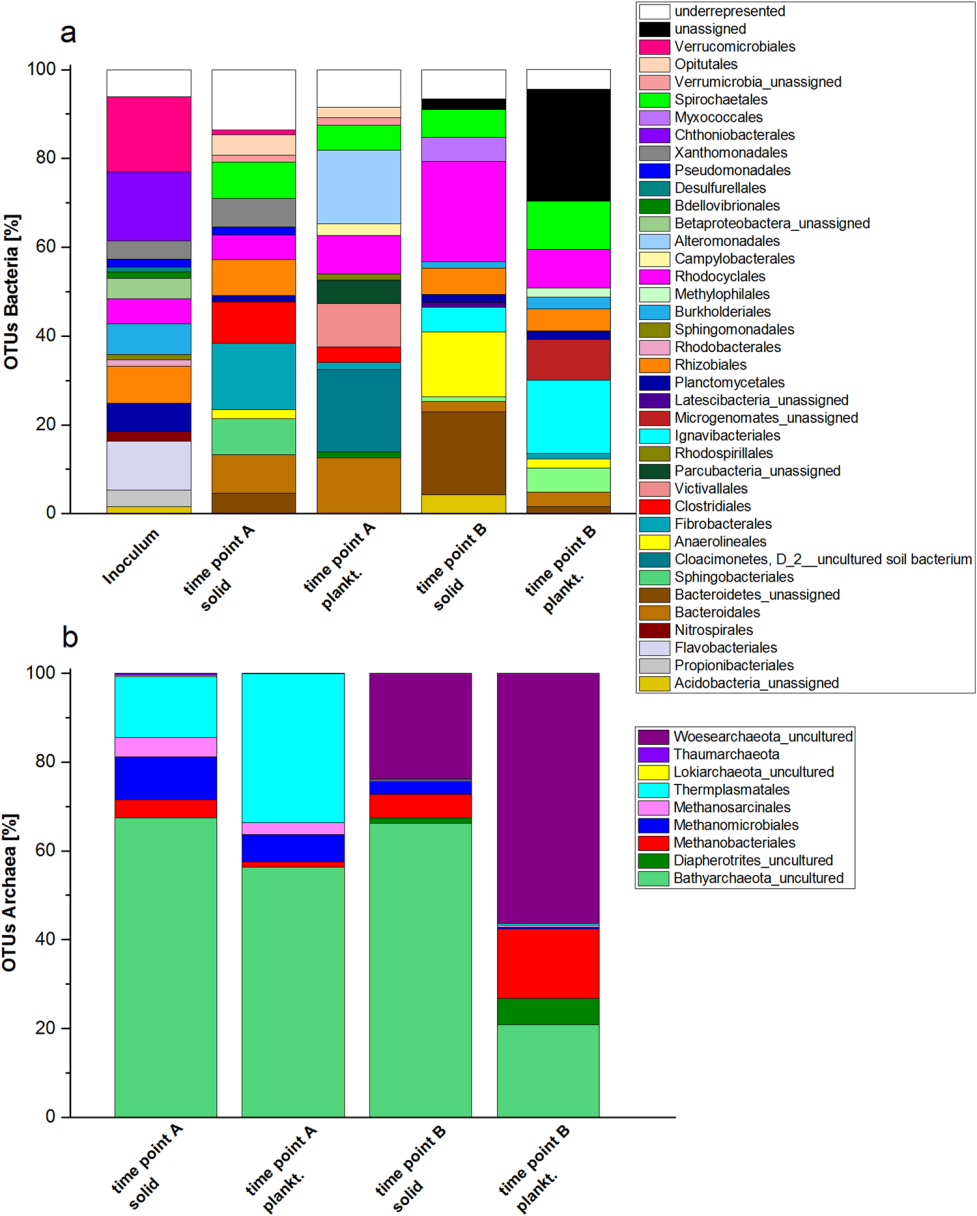
Microbial diversity in the laboratory reactors and inoculum. 16S rRNA gene amplicon analysis and the relative abundance of microbial communities in the inoculum and wood chip reactors for two time points: time point A low and time point B high nitrate concentrations. (**a**) Percentage of OTUs for bacteria. Only OTUs >1% of the total number of amplicons are shown. (**b**) Percentage of OTUs for archaea. All OTUs are shown. As a reference database, the SILVA 16S v 128 97% was used.

Only a very small number of sequence reads obtained were assignable to the archaea from the inoculum sample. Since the significance of this sequencing result is probably minimal, we did not include this data in Fig. 5b.

### Metatranscriptomic analysis

The metatranscriptome of the wood chip reactors was analyzed to uncover the potential producers of key proteins involved in cellulose hydrolysis, nitrate elimination and the unexpected ammonium production under high nitrate conditions (time point B). Hence, the phylogenetic information within the assembled metatranscriptome reads was compared to the 16S rRNA gene amplicon analysis to identify potential key players. A total of 669,975,316 bp were assembled into 2,806,808 contigs.

**Figure 6:**
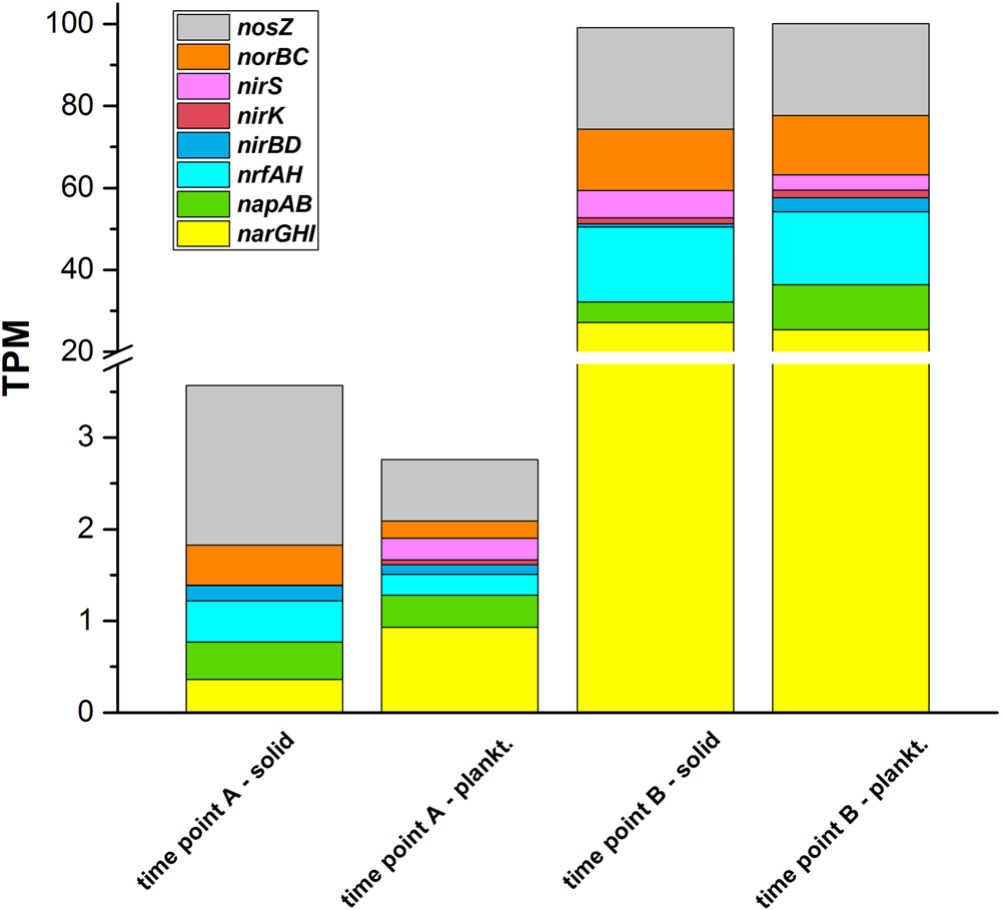
Total number of TPM for genes involved in nitrate elimination. Total numbers of TPM for all genes encoding the periplasmic nitrate reductase (NAP), respiratory nitrate reductase (NAR), dissimilatory nitrite reductase (NIR), ammonium producing nitrite reductase (NRF), nitric oxide reductase (NOR), and nitrous oxide reductase (N2OR) for the planktonic and solid samples, as well as for low (time point A) and high nitrate concentrations (time point B).

In general, the Transcripts Per Million (TPM) values for typical genes involved in the nitrate removal processes (*nar*, *nir*, *nor*, *nos*, *nap*, *nfr*) increased from time point A (low) to time point B (high nitrate concentrations). This suggests that the expression of these genes was induced at higher nitrate concentrations (Fig. 6). The ratio between the TPM values of time point A and time point B were compared to quantify the level of induction (Table 1).

**Table 1:** Ratio of TPM values for key genes of nitrate reduction between time point A (low nitrate concentration) and time point B (high nitrate concentration).

	nrfAH	napAB	narGHI	nirS	nirK	nirBD	norBC	nosZ
Biofilm	40.7	12.1	75.5	1269.5	—	4.8	33.9	14.23
Planktonic cells	77.5	31.2	27.4	15.7	35.2	33.2	76.3	33.4

The TPM values were compared for samples from the planktonic and biofilm phase. Transcripts of the *nirK* genes were not detectable at time point A in the biofilm sample. Therefore, it was not possible to calculate a ratio for the induction of this gene within the biofilm.

Besides typical genes for enzymes involved in denitrification, also the expression of *nrf* genes increased from time point A to B (Table 1). The *nrf* gene encodes a nitrite reduction enzyme, which reduces nitrite into ammonium.

**Figure 7:**
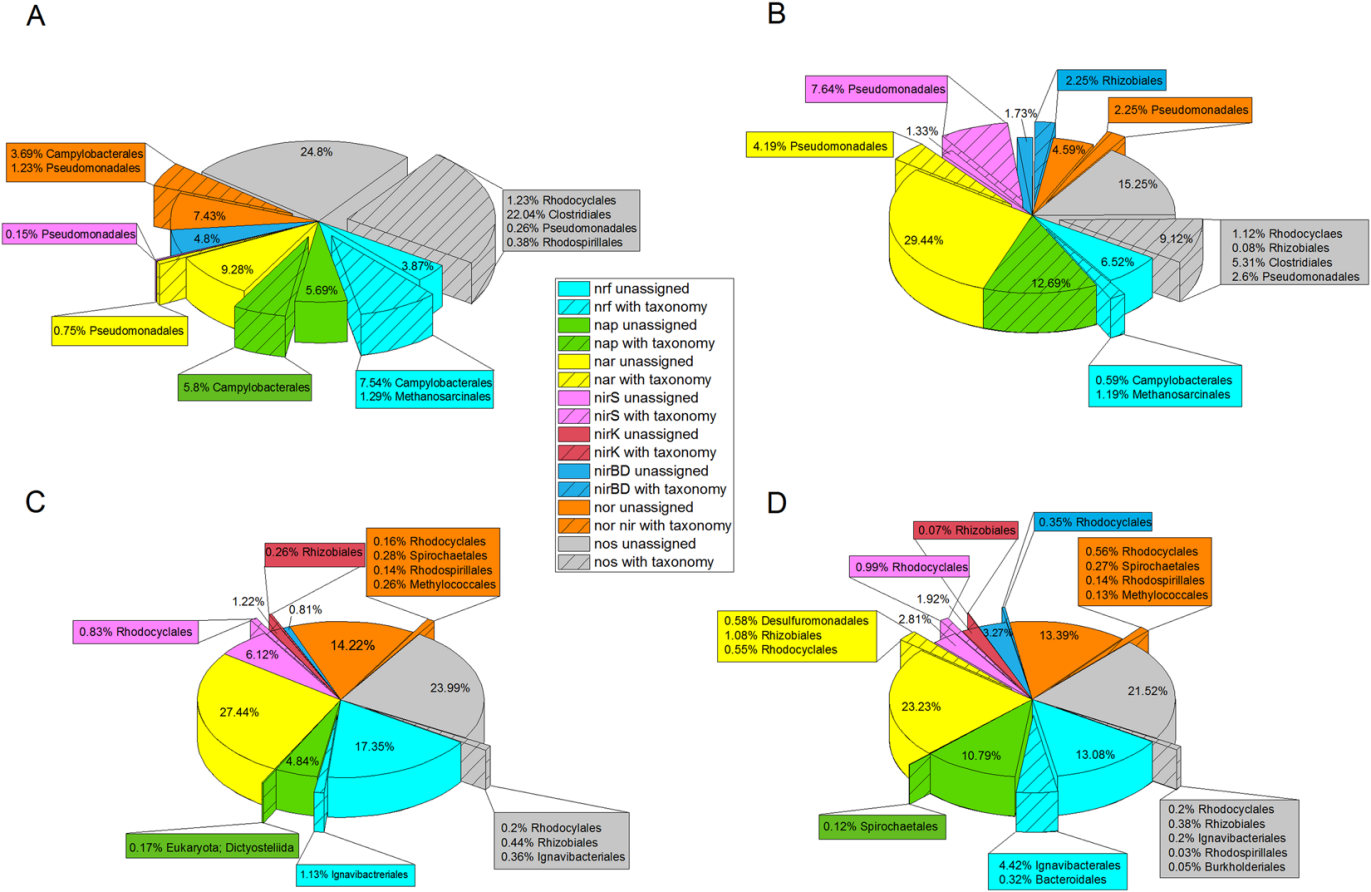
Relative abundance of gene expression for typical genes involved in denitrification and DNRA and their taxonomic assignment. The taxonomic identification for the transcribed genes could be assigned only for subsets of the overall number of reads. (**A**) Wood chip solid phase sample with a low nitrate concentration (time point A). (**B**) Wood chip planktonic phase sample with a low nitrate concentration (time point A). (**C**) Wood chip solid phase sample with a high nitrate concentration (time point B). (**D**) Wood chip planktonic phase sample with a high nitrate concentration (time point B).

Moreover, the majority of reads for the *nrf* gene that could be taxonomically assigned to a bacterial order clustered within the Ignavibacteriales (Fig. 7C–D), which appeared in the 16S rRNA gene amplicon analysis only for time point B (high nitrate conditions) and could not be detected before. For time point A *nrf* genes that could be taxonomically linked clustered in the order Campylobacterales.

Of note, the metatranscriptomic analysis revealed that genes involved in the denitrification process (*nar*, *nir*, *nor*, *nos*) were probably not expressed by similar organisms under low (time point A) and high (time point B) nitrate concentrations. For low nitrate concentrations (Fig. 7A,B), taxonomically linked orders clustered mainly within the Pseudomonadales. No mRNAs of denitrification genes could be assigned to this order under high nitrate concentrations. Here, the assignable mRNAs were taxonomically linked mainly to the order Rhodocyclales and Rhizobiales. Nevertheless, for most of the reads, a taxonomic assignment was not possible. However, most of these organisms that could be taxonomically linked and were likely involved in the nitrate elimination process belonged to the phylum Proteobacteria (except Clostridiales, Ignavibacteriales, and Spirochaetales). This suggested that members of this order may function as key players in denitrification beds. The distribution of organisms involved in the cellulose degradation process was different. Here, members that could be taxonomically linked belonged to several different phyla, including Firmicutes, Actinobacteria, Proteobacteria, Bacteroidetes, and Spirochaetes.

In the degradation of cellulose mainly three different types of enzymes are involved - endoglucanases (endo-1,4-ß-glucanase) (EC: 3.2.1.4), exoglucanases (exo-1,4-ß-glucanase) (EC 3.2.1.74) or cellobiohydrolases (EC 3.2.1.91) as well as ß-glucosidases (cellobiases) (EC 3.2.1.21)^27^. Interestingly, the TPM values for transcripts of genes that code for endoglucanases increased from 6.78 to 73.8 for the solid phase and 10.03 to 52.66 for the planktonic phase. Those reads, which could be taxonomically linked, clustered mainly in the phyla Firmicutes (order Clostridiales), Proteobacteria and Actinobacteria (order Actinomycetales). Expression of genes from Actinomyctelales could only be detected for high nitrate concentrations (time point B), especially in the solid phase (TPM value of 2.36 compared to 0.44 in the planktonic phase). In contrast, transcripts that were linked to Clostridiales could be found for low (TPM value 0.06 for the solid phase, 0.5 for the planktonic phase) and high nitrate concentrations (0.43 for the solid phase and 0.44 for the planktonic phase), even though the Clostridiales were underrepresented in the 16 rRNA gene amplicon analysis for time point B. Compared to endoglucanases, exoglucanases were significantly less expressed under low (0.13 for solid and 0.09 for planktonic phase) and high nitrate conditions (1.17 for the solid and 0.89 for the planktonic phase). Transcripts of genes that code for ß-glucosidases, which degrade cellobiose to glucose, showed again an increased expression at time point B. Here, the sum of TPM values was higher in the solid phase and increased considerably from low (49.26 for the solid and 32.09 for the planktonic phase) to high nitrate concentrations (1263.82 for the solid and 101.76 for the planktonic phase). Transcripts that could be taxonomically identified were expressed in different organisms compared to the endoglucanases as they were mainly found to cluster within the Bacteroidetes (0.57 solid and 0.3 planktonic for time point A and 0.95 solid and 2.16 planktonic for time point B).

We observed that the TPM values for the key enzyme for methanogenesis, the methyl-coenzym M reductase (MCR) (which includes the subunits α, β, and γ) increased considerably with higher nitrate concentrations. The sum of the TPM values for samples of the solid phase increased from 5.08 for time point A to 48.6 for time point B. This trend was even more pronounced for the samples of the planktonic phase. Here, the total sum of TPM increased from 2 to 99.2. This result correlated well with the observed methane production after the addition of higher nitrate concentrations. The detected mRNAs could be taxonomically linked to members of the orders Methanosarcinales, Methanobacteriales, and - to a minor extend - Methanomicrobiales. All orders could also be found in the archaeal 16S rRNA gene amplicon analysis. Hydrogen and carbon dioxide can be used as substrate for methanogenesis by the organisms belonging to the detected orders. To determine the potential source of hydrogen, we analyzed the TPM values for all hydrogenase transcripts of the metatranscriptome and aimed to elucidate which organisms were expressing these genes. Overall, the TPM values for all hydrogenases increased again with higher nitrate concentrations (from 316.72 to 780.5 for the solid phase and from 242.88 to 490.9 for the planktonic phase). Excluding the Archaea, the main reads that could be taxonomically assigned belonged to different bacterial orders when comparing time point A (low) and time point B (high nitrate concentrations). Members of orders that express more hydrogenases under low nitrate concentrations belonged preferentially to Clostridiales and Spirochaetales. Under high nitrate concentrations, the proportion of the taxonomically linked reads shifted towards members of the order Rhodocyclales, Ignavibacteriales, Methylococcales, and Rhizobiales. All of these orders, except for the Methylococcales, increased or appeared for the first time after the addition of higher nitrate concentrations (time point B). Detailed information regarding the number of TPMs can be found in the supplementary information (Tables S6–S8).

## Discussion

For the first time, this study analyzed the phylogenetic and metabolic diversity in denitrification beds following laboratory simulation, which could be seen as a synthetic microbial ecosystem. De Roy *et al*. defined this as a rationally designed ecosystem with controlled conditions, meaning that it has less complexity, more controllability, and limiting influencing factors. It also allows the observation of evolving parameters and their consequences^28^. Here, this study could be seen as a representation of a naturally occurring environment, which used batch systems to simulate field denitrification beds. One could question the applicability of these results to a field flow-through system. Nevertheless, 1 year ago, we started to analyze the same processes in field denitrification beds of a scale of 214 m^3^. Throughout the year, we observed these beds for long periods of time (3 months) and found that the flow through the system was very low to almost undetectable. Hence, even denitrification beds in the field have a batch-like character for at least a considerable part of the year.

The developed reactors showed a stable and continuous nitrate reduction for moderate nitrate concentrations up to 6 mmol L^−1^. Nevertheless, it seems that higher nitrate concentrations above 11 mmol L^−1^ could be a challenge for the functionality of these systems. These high nitrate pulses were chosen to represent a stress test for the denitrification bed and to demonstrate the nitrate elimination capacity under different loading rates.

We observed that under higher nitrate concentrations, a shift from denitrification to dissimilatory nitrate reduction to ammonium occurred. This does not support common textbook knowledge, which assumes that DNRA is sustained by a high C_org._/NO_3_^−^ ratio^17^. Members of the order Ignavibacteriales seem to be key players in the observed shift from denitrification to DNRA. Bioinformatic data from the available genome of *I*. *album* suggest that this organism can catalyze the second step of DNRA; namely, the reduction of nitrite to ammonium, because it possesses a NrfAH complex. However, it is unable to convert nitrate to nitrite^29^. Nevertheless, the *I*. *album* genome also contains genes for a nitric oxide and a nitrous oxide reductase, which suggests that it could also participate in the denitrification process^29^. It is currently unknown under which conditions *I*. *album* chooses the ammonium production pathway and when it catalyzes the reduction of NO to N_2_O and, subsequently, to N_2_. Still, *Ignavibacterium* is dependent on other nitrate reducing organisms that catalyze the first nitrate reduction steps. The high level of microbial diversity in the laboratory reactors indicates that a complex network of organisms participate in the nitrate reduction process. Some organisms, such as *Ignavibacterium*, participate only in some steps of denitrification or the DNRA process. Others, such as members of the order Pseudomonadales and Rhodocyclales, possess and express all genes involved in the denitrification process. Nevertheless, members of Pseudomonadales (mainly genus *Pseudomonas*; Table S9) seem to play a relevant role in denitrification at low nitrate concentrations, while Rhodocyclales and Rhizobiales apparently predominate at higher nitrate conditions.

The presence of members of the Planctomycetales in nearly every sample could suggest a potential involvement of anammox bacteria in the nitrate elimination process. Still the sequences that could be assigned to a genus do belong to members of the *Planctomyces* and *Gemmata*. Members of this genus are not known to conduct the anammox process. Also, the metatranscriptomic analysis did not reveal the expression of a hydrazine oxidoreductase (HZO), the key enzyme for anammox bacteria^30^. An absence of anammox organisms would also be expected due to a higher organic carbon content in the studied denitrification bed systems, because Winkler *et al*. reported on an inhibition of these organisms in environments with COD/N ratio higher than 0.5^31^.

We observed that even the process of cellulose degradation was positively influenced by higher nitrate loading rates as indicated by the higher TPM values for genes encoding cellulose degrading enzymes. Nevertheless, the metatranscriptomic analysis suggests that both processes are only to some extend catalyzed by the same organisms. Hence, via a so far unknown mechanism cellulose degraders belonging to the Clostridiales, Actinomycetales or Bacterioidetes seem to contribute from higher nitrate loading rates. The higher activity of these organisms will result in a positive feedback on denitrifying organisms that feed on typical fermentation end products. The here described transcriptomic results corroborate a study by Berlemont and Martiny (2013). They describe lineages expressing only genes for ß-glucosidases as potential opportunists, because they thrive on cellobiose, which is produced by other organisms that catalyze the initial steps of cellulose hydrolysis^32^. They find a higher proportion of potential opportunists in the Bacteroidetes and more potential cellulose degraders within the Proteobacteria, Actinobacteria and Firmicutes^32^. The same is valid for this study in which the Bacteroidetes mainly produce ß-glucosidases while Actinobacteria and Proteobacteria seem to be the main producers of enzymes catalyzing the initial depolymerization of cellulose.

The gas analysis revealed detectable CO_2_ concentrations beginning on day 112 even though the TOC concentration steadily decreased after day 20. A malfunction of the sensors before day 112 seems to be unlikely due to the regularly conducted calibration and inspection of the sensors, but cannot be totally excluded. Nevertheless, a later occurrence of CO_2_ could be the result of the solubility of CO_2_ in the medium and the headspace volume of the reactor. The TOC decreased from around 400 mg L^−1^ to 120 mg L^−1^ at day 112, meaning that the overall volume produced equaled approximately 940 ml CO_2_. Nevertheless, the solubility of CO_2_ in water is quite high (1.7 g L^−1^ at 20 °C). Hence, a large amount of the CO_2_ likely dissolved in the medium first. This hypothesis is corroborated by the measured concentrations of inorganic carbon, which increased from around 60 mg L^−1^ at the beginning of the experiment to around 250 mg L^−1^ at the start of the gas detection until 500 mg L^−1^ in a later stage of the experiment.

Interestingly, methane was also detectable only during the later stages of the experiment. More specifically, its detection coincided with the addition of high nitrate concentrations. Here, the reason for this cannot be the solubility of methane, since it is 0.02 g L^−1^ at 20 °C (85-fold lower than CO_2_). Using 16S rRNA gene amplicon sequencing, methanogens were detectable at time point A and time point B. Nevertheless, the higher activity of the methanogens, which was suggested by the metatranscriptomic analysis, correlated with higher nitrate concentrations (time point B). This result was unexpected because denitrification should hamper methanogenesis as a result of the higher availability of Gibb’s free energy ΔG^0’^ ^33,34^. Even if there were a sufficient amount of substrate for both processes, other studies showed that the intermediates of the denitrification process seem to have toxic effects on methanogenic organisms^35^. The high internal porosity of the wood chips could be a possible explanation for the presence of methanogens. Thick biofilms within the wood chips could have provided a micro-niche with low concentrations of toxic intermediates. That methanogenesis and denitrification could occur simultaneously is corroborated by several examples of bioreactors in which both processes are catalyzed at the same time^36^. Moreover, in this study, the increased number of TPM for hydrogenases supports the hypothesis that methanogenesis could be feasible under high nitrate conditions because of an increased amount of H_2_ as a substrate. Nevertheless, it has not yet been shown that high nitrate doses can shift the flux in an ecosystem towards methanogenesis. In future studies, we will attempt to understand the reason for the intervals of gas production that we detected, which proved to be reproducible in a separate experiment under similar conditions (data not shown).

Generally, it is essential when installing a denitrification bed to consider and constantly observe factors which can cause a collateral damage to the environment as for instance the production of greenhouse gases like N_2_O or CH_4_ or the release of toxic NO_2_^−^ due to an incomplete denitrification process. Reducing nitrate emissions to freshwater systems should not result in a contra-productive outcome of producing large quantities of greenhouse gases or other toxic side products.

In summary, this study shows for the first time the fundamental basis for nitrate elimination processes in denitrification beds. Moreover, this study revealed new facets of the nitrogen cycle and its interplay with the carbon cycle, which should be considered for a number of ecosystems besides denitrification beds.

## Methods

### Laboratory bioreactors

Three 2.4 L glass reactors were filled with untreated poplar wood chips as carbon sources (in triplicate). The carbon source was added to the reactors to a bed width of 4 cm and compacted using autoclaved stones. A PTFE (polytetrafluoroethylene) tube fixed in the reactor wall served as an access point for nitrate addition and sampling, and the top outlets of the reactors were directly connected to IR-based gas sensors (Bluesens, Herten, Germany). This allowed the determination of CO_2_ and CH_4_ concentrations in the gas phase. The detection limits of the sensors were 50 ppm (2.2 mmol/m^3^) for CO_2_ and 400 ppm (16.6 mmol/m^3^) for CH_4_. Figure 1 illustrates the laboratory reactor setup.

The bioreactors were loaded with artificial moor media (Table S1) containing 1.18 mmol L^−1^ KNO_3_. The bioreactors were then inoculated with 1% drainage water from the study area called *Mürmes*, which is located in the Vulkaneifel (Germany) and is influenced by nitrate-rich water from agricultural drainage. The starting concentration of nitrate (1.18 mmol L^−1^) for the experiment corresponded to the highest nitrate concentration measured in December 2014 at one of the drainage sites. The final volume equaled 1.8 L in all reactors, and the reactors were placed on a rotary stirrer and mixed with 125 rpm/min. The reactors were incubated at room temperature and in the dark to avoid algae growth. The pH was observed regularly and remained stable between 7 and 7.5.

### Sampling and analytical analysis

Samples from the laboratory bioreactors (5–7 ml) were collected two to three times per week over a period of 200 days. Samples were filtered through a 0.2 µm PTFE filter and analyzed for NO_3_^−^, NO_2_^−^, and NH_4_^+^ using a spectral photometer DR3900 and cuvette tests (Hach) according to the manufacturer’s guidelines. The detection limits for NO_3_^−^, NO_2_^−^, and NH_4_^+^ were 0.02 mmol L^−1^, 0.001 mmol L^−1^, and 0.001 mmol L^−1^, respectively. Total organic carbon (TOC) levels of unfiltered, diluted water samples were determined using a TOC-analyzer (Multi N/C 2100 S Analytic Jena, Germany). A standard of 1 g L^−1^ TOC was analyzed with every measurement to assure the quality of the measurements. Organic carbon compounds, particularly organic acids, were analyzed via HPLC according to Kipf *et al*.^37^.

Water spiked with KNO_3_ was added to the reactors when the NO_3_^−^ concentration decreased below 0.24 mmol L^−1^. This was done in order to achieve the initial NO_3_^−^ concentration of 1.18 mmol L^−1^. After a few cycles of NO_3_^−^ addition, the NO_3_^−^ concentration was increased by the two-fold (2.36 mmol L^−1^), five-fold (5.9 mmol L^−1^), ten-fold (11.8 mmol L^−1^), and twenty-fold (23.6 mmol L^−1^).

On day 62 (time point A) and day 177 (time point B), all reactors were opened and the carbon substrate from the center of the reactors and the planktonic phase from between the wood chips were sampled. These samples served as starting materials for the extraction of DNA and RNA.

### DNA and RNA extraction

Genomic DNA was extracted from a 1 ml liquid sample or from a 100–250 mg sample of wood chips using the innuSPEED Soil DNA Kit (Analytic Jena) or with the Wizard Genomic DNA Purification Kit (Promega) according to the manufacturer’s guidelines. DNA was extracted from the planktonic or solid phase of the wood chip reactors and from the drainage water (inoculum).

Samples were obtained from the wood chip reactors and treated with RNA Protect Solution (Qiagen) prior to storage at –80 °C. RNA was isolated using the InnuSPEED Bacteria/Fungi RNA Kit (Analytic Jena) according to the manufacturer’s guidelines. The remaining DNA was removed using the DNA-free Kit (Ambion). Afterwards, the RNA samples were tested for remaining genomic DNA by using them as a template for PCR reactions with the universal 16S rRNA gene primers 27F and 1525R^38^. While control reactions with samples prior to DNase treatment showed the expected amplified 16S rRNA genes, the samples after treatment did not lead to an amplified DNA fragment.

### 16S rRNA gene amplicon sequencing and analysis

The phylogenetic composition of the biofilm present on the wood chips and the planktonic communities in the sample and inoculum was analyzed using 16S rRNA gene amplicon sequencing. Because the activity of the three independent reactors was similar, the DNA of the corresponding triplicates from the wood chip reactors was pooled, ensuring that identical amounts of DNA from each sample were added. Fragments of the 16S rRNAs were amplified using primers Bact_341F and Bact_805R for bacterial genes and A519F and U906R for archaeal genes^39,40^. Detailed information of the amplicon sequencing results according to Field *et al*. (2008) can be found in the supplementary section (Table S4). Sequencing was conducted by IMGM Laboratories GmbH (Martinsried, Germany) on an Illumina MiSeq platform using 2 × 250 bp paired-end (PE) reads. Signal processing, de-multiplexing, and trimming of adapter sequences were conducted by IMGM Laboratories with the MiSeq Reporter 2.5.1.3 software. Further bioinformatic analysis of the 16S rRNA gene amplicon sequencing (primer cutting, quality and length trimming, merging, OTU clustering, and phylogenetic analysis) was conducted using the CLC Genomic Workbench software 10.0.1 with the additional microbial genomic module 2.0. Results were exported in BIOM format^41^ and finally analyzed with the R package DESeq. 2^42^ v3.5 to detect differentially abundant taxa between samples of low and high nitrate concentration. In the analysis only results with BH adjusted p-values < 0.05 were considered. Table S2 provides an overview of the sequencing analysis parameters used and the results for each sample.

### Metatranscriptome sequencing and analysis

For the metatranscriptomic analysis, the RNA triplicate samples of the biofilm and planktonic phases were pooled again as described for 16S rRNA gene amplicon sequencing. The metatranscriptome was sequenced on an Illumina MiSeq sequencer using 2 × 100 bp PE reads. This analysis was performed either at the sequencing facility of the Karlsruhe Institute of Technology or the IMGM Laboratories GmbH (Martinsried, Germany) (Table S3).

All metatranscriptomic reads were assembled using MEGAHIT^43^ v1.1.1 in its most sensitive mode, iterating over *k*-mer sizes of 21, 31, 41, 51, 61, 71, 81, 91, and 99 to reconstruct full-length genes. Subsequently, Transcripts Per Million (TPM) were calculated using Kallisto^44^ v0.43.1. A functional annotation of the assembled genes was performed using GhostKOALA^45^ and Paladin^46^ v1.3.1. Taxonomic assignments were carried out using the BLASTN-based taxonomic binning algorithm implemented in taxator-tk^47^ v1.3.3.

### Data availability

All data generated or analyzed during this study are included in this published article (and its Supplementary Information files). All DNA and RNA sequences that were retrieved for this study are publicly available through NCBI BioProject PRJNA383490. BioSample accessions: SAMN07728356, SAMN07728357, SAMN07728358, SAMN07728359, SAMN07728360.

## Electronic supplementary material

Supplementary Material, Figure S1-S2, Table S1-S4

Supplementary Table S5

Supplementary Table S6

Supplementary Table S7

Supplementary Table S8

Supplementary Table S9
