# Supplementary material for: Investigation of different nitrogen reduction routes and their key microbial players in wood chip-driven denitrification beds
Source: Sci Rep. 2017 Dec 5;7:17028. doi: 10.1038/s41598-017-17312-2 (PMC5716999; doi:10.1038/s41598-017-17312-2)
Supplement: Supplementary file 1 — Supplementary Material, Figure S1-S2, Table S1-S4 [file 41598_2017_17312_MOESM1_ESM.doc]

**Investigation of different nitrogen reduction routes and their microbial key players in wood- chip driven denitrification beds**

Victoria Grießmeier^1^, Andreas Bremges^2,3^, Alice C. McHardy^2^, and Johannes Gescher* ^1,4^

^1^Department of Applied Biology, Institute for Applied Biosciences, Karlsruhe Institute of Technology (KIT), Karlsruhe, Germany

^2^Computational Biology of Infection Research, Helmholtz Centre for Infection Research, 38124 Braunschweig, Germany

^3^German Center for Infection Research (DZIF), partner site Hannover-Braunschweig, 38124 Braunschweig, Germany

^4^Institute for Biological Interfaces, Karlsruhe Institute of Technology (KIT), Eggenstein-Leopoldshafen, Germany

Corresponding author:

Prof. Dr. Johannes Gescher

Karlsruhe Institute of Technology
Institute of Applied Biosciences
Department of applied biology
E-mail: [johannes.gescher@kit.edu](mailto:johannes.gescher@kit.edu)

## Supplemental material

In the following the supplementary data mentioned in this article are shown:

| **A** | **B** |
| --- | --- |
| 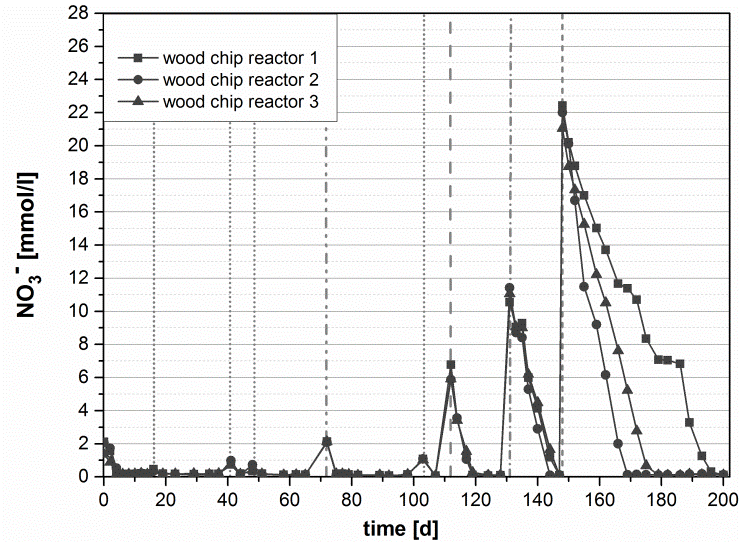 | 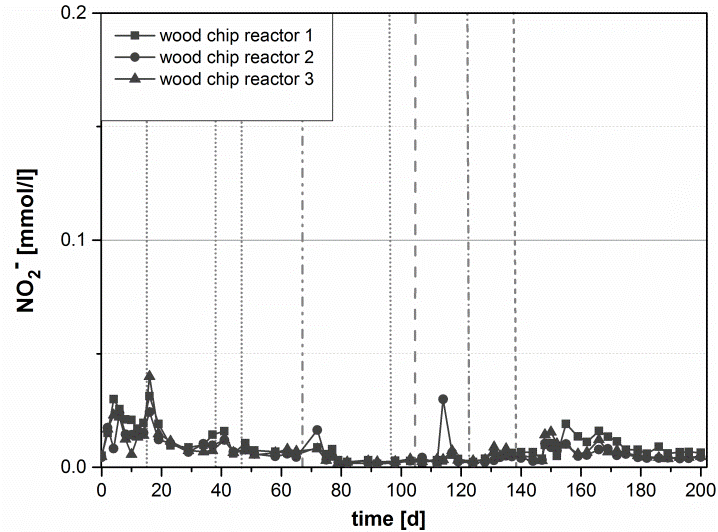 |
| **C** |  |
| 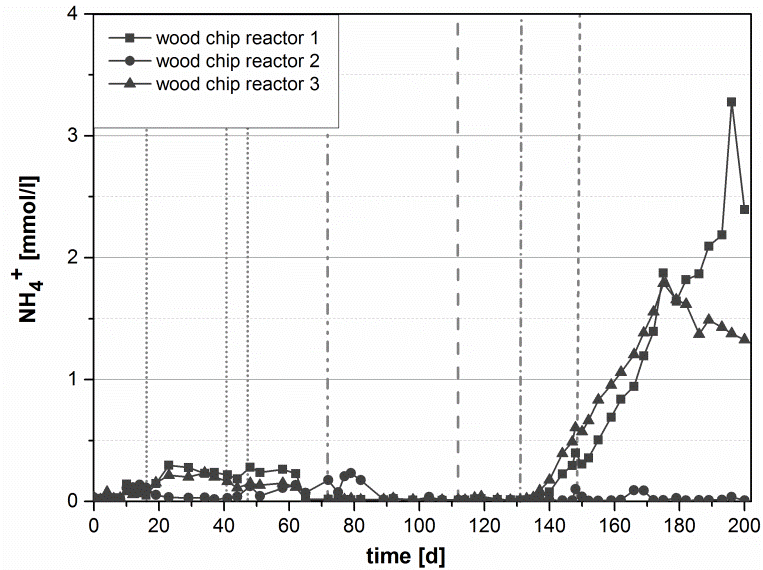 |  |

Figure S1: Detailed analysis of the triplicate concerning nitrate (A), nitrite (B) and ammonium (C) during the runtime of the laboratory denitrification filled with wood chips. Dashed vertical lines symbolize different amounts of nitrate additions:
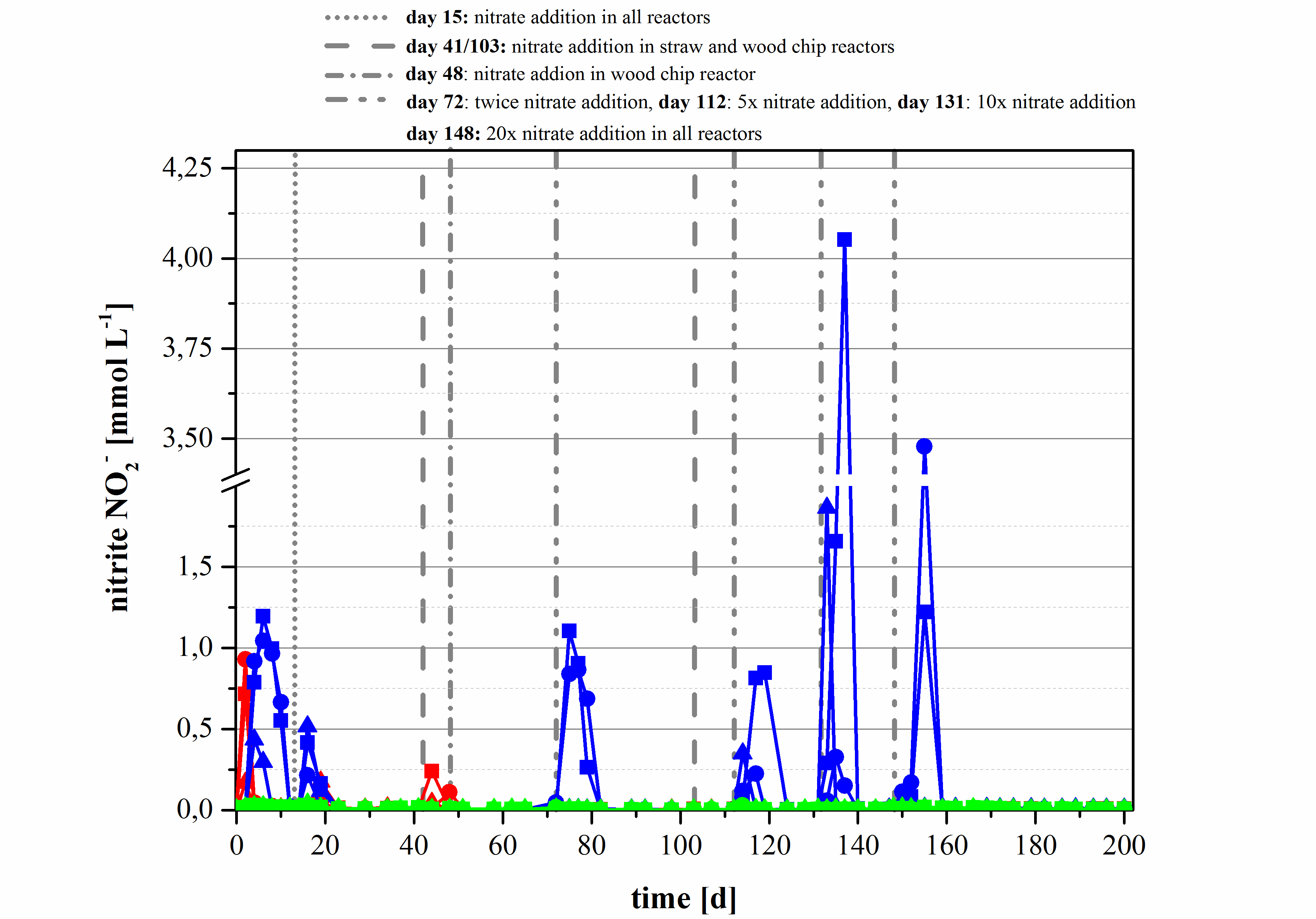
nitrate addition to all reactors at Day 15/41/48/103,
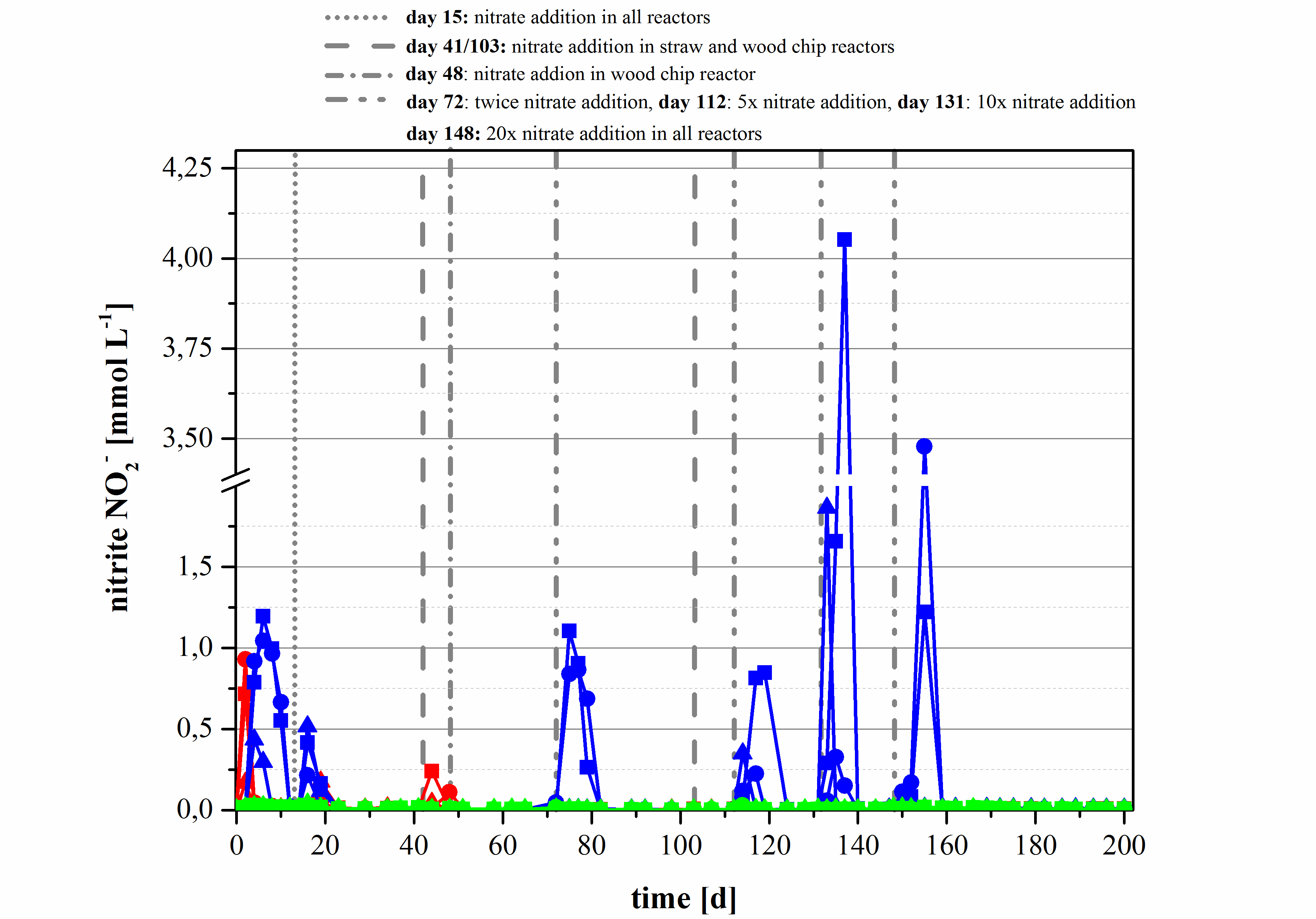
double nitrate addition at Day 72, 5x nitrate addition at Day 112, 10x nitrate addition at day 131, 20x nitrate addition at Day 148 in all reactors.

| **A** | **B** |
| --- | --- |
| 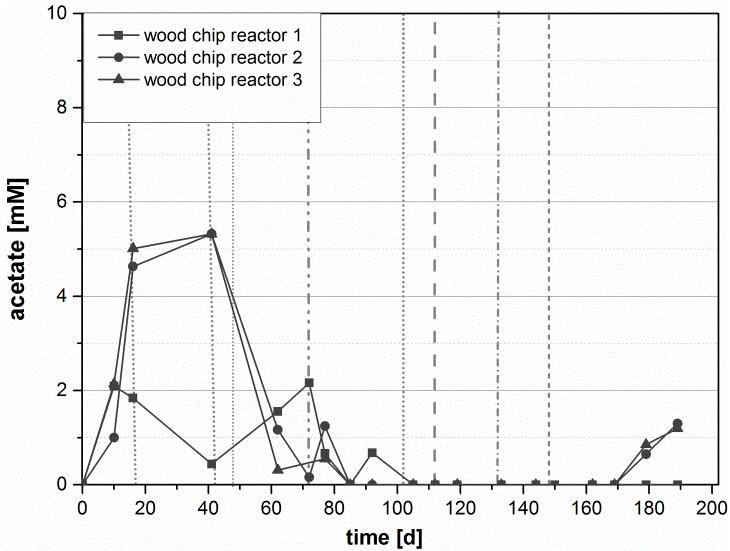 | 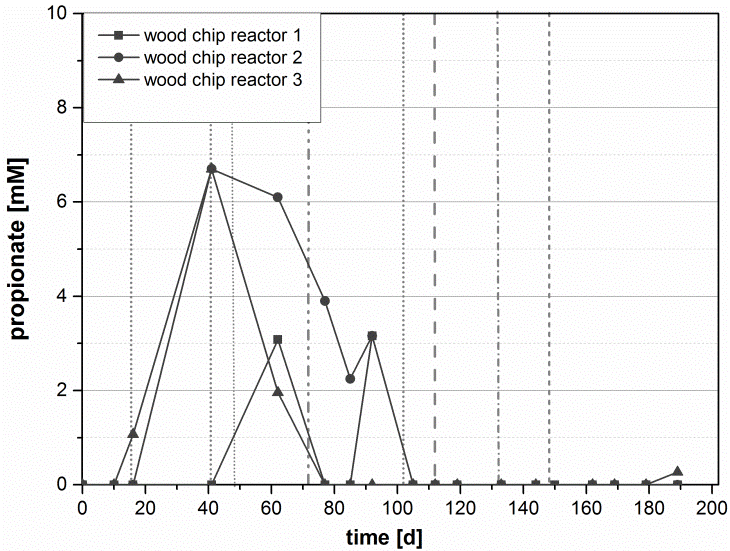 |
| **C** |  |
| 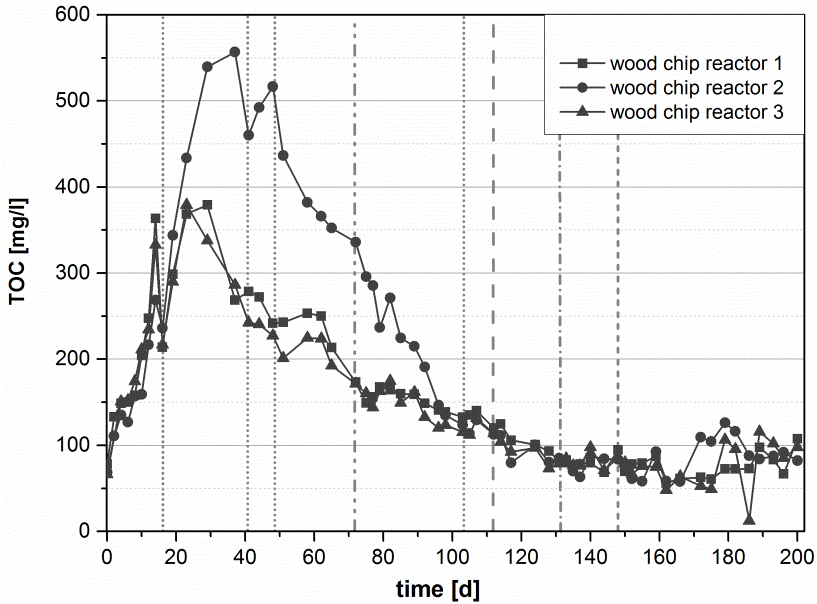 |  |

Figure S2: Detailed analysis of the triplicate concerning propionate (A), acetate (B) and the total organic carbon TOC (C) during the 200 days runtime of the reactors filled with wood chips. Dashed vertical lines symbolize different amounts of nitrate addition:
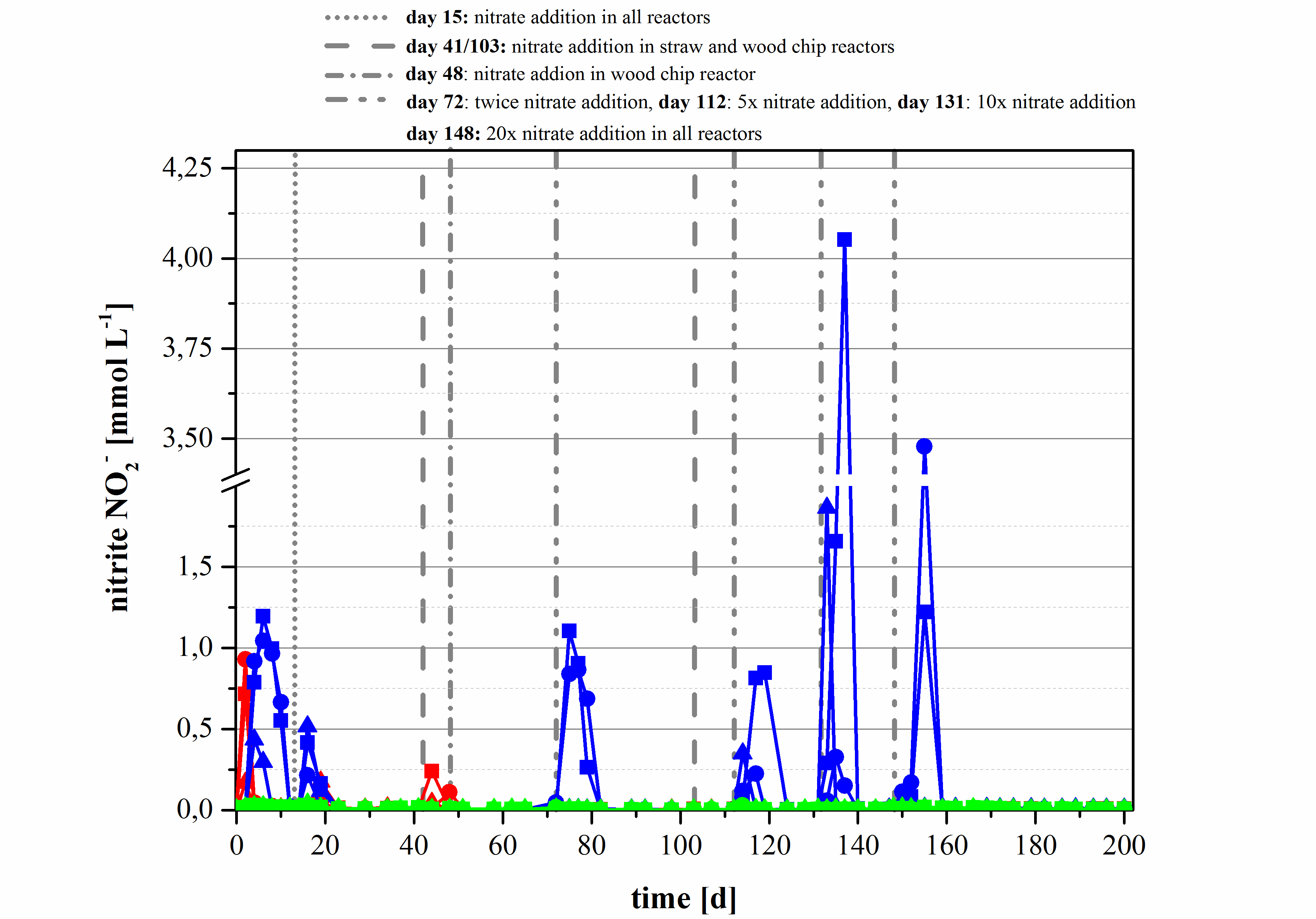
nitrate addition to all reactors at Day 15/41/48/103,
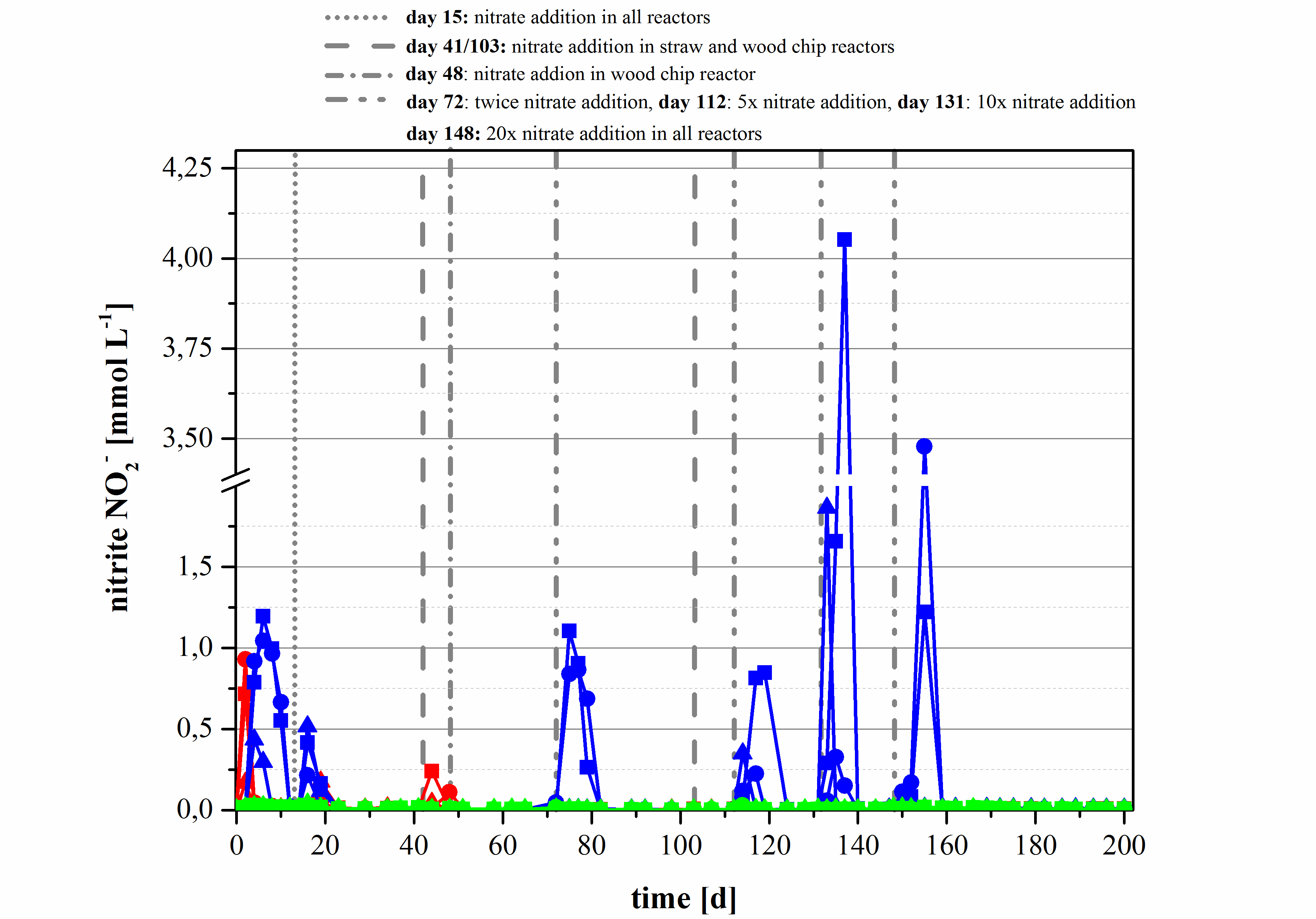
double nitrate addition at Day 72, 5x nitrate addition at Day 112, 10x nitrate addition at day 131, 20x nitrate addition at Day 148 in all reactors.

Table S1: Composition of the synthetic moor media based on the analysis of Fors*t et a*l., 1997. pH was adjusted to 6.8 based on the first analysis from Deccember 2014.

| **Substance** | **Concentration per liter** |
| --- | --- |
| CaCl_2_ x H_2_O | 0.49 mM |
| MgSO_4_ x H_2_O  K_2_HPO_4_  NH_4_Cl | 0.39 mM  0.0005 mM  0,018 mM |
| KNO_3_ | 4.9 mM |

Table S2: Number of raw, merged and trimmed reads and number of reads in OTUs after phylogenetic analysis. SILVA 16S v 128 97% was used as reference database.

| **Sample** | **No. of reads** | **No. of merged reads** | **No. of reads after trim** | | **Avg. length after trim** | **Reads in OTUs** |
| --- | --- | --- | --- | --- | --- | --- |
| Inoc_Bac | 248'562 | 172'810 | | 84'578 | 402 | 24’354 |
| Time point A, solid; Bac | 293'988 | 222'884 | | 108'705 | 402 | 22’482 |
| Time point A, plankt.; Bac | 476'872 | 418'006 | | 205'505 | 402 | 76'513 |
| Time point B, solid; Bac | 238'994 | 181'666 | | 89'862 | 402 | 18'138 |
| Time point B, plankt.; Bac | 161'298 | 125'554 | | 48'909 | 402 | 9'082 |
| Inoc_Arch | 304'034 | 50'714 | | 22'829 | 364 | Not passed, because of low coverage |
| Time point A, solid; Arch | 366'724 | 166'772 | | 80'522 | 364 | 63'347 |
| Time point A, plankt.; Arch | 243'088 | 136'514 | | 65'872 | 364 | 57'127 |
| Time point B, solid; Arch | 302'298 | 235'902 | | 117'458 | 364 | 86'199 |
| Time point B, plankt.; Arch | 126'590 | 83'148 | | 41'484 | 364 | 24'243 |

Table S3: Sequencing and assembly statistics for the metatranscriptome.

| **Sample** | **No. of reads (contigs)** | **Total bases** |
| --- | --- | --- |
| S1 – time point A solid (low NO_3_^-^) | 258,336,354 | 25,833,635,400 |
| S2 – time point A plankt. (low NO_3_^-^) | 301,169,654 | 30,116,965,400 |
| S3 – time point B plankt. (high NO_3_^-^) | 86,245,656 | 11,445,354,745 |
| S4 – time point B solid (high NO_3_^-^) | 47,085,066 | 6,219,451,978 |
| Metatranscriptome assembly | 2,806,808 | 669,975,316 |

**Table S4:** Information on amplicon and metatranscriptomic sequencing.

| **NCBI project ID** | PRJNA383490 |
| --- | --- |
| **Accession Number** | SAMN07728356, SAMN07728357, SAMN07728358, SAMN07728359, SAMN07728360 |
| **Taxonomic group** | Environmental sequencing project; Archaea and Bacteria |
| **Source of the material A (drainage water)** |  |
| geographical location | Vulkaneifel |
| latitude | 59° 9' N |
| longitude | 6° 53' E |
| depth | Surface water |
| time of sample collection | 2014-12-08 |
| sample type | Agricultural drainage water |
| **Source of the material B (enrichments in laboratory wood chip based denitrification systems)** |  |
| geographical location | Karlsruhe Institute of Technology |
| sample type | Water and wood chip samples |
| time of sample collection | 1. 2015-04-15 2. 2015-08-08 |
| habitat type | Agricultural drainage water |
| **Isolation and growth conditions** | Source material A: environmental sample  Source material B: Batch denitrification systems. Wood chips served as carbon and electron source, nitrate as electron acceptor. Reactor were incubated in the dark at room temperature on a stirrer (125 rpm) |
| **Volume of samples** | 100-250 mg of wood chips  1 ml of liquid sample |
| **DNA and RNA extraction method** | DNA isolation: InnuSPEED Soil DNA Kit, Analytic Jena  RNA isolation: InnuSPEED Bacterial/Fungi RNA Kit, Analytic Jena |
| **Sequencing method** | DNA: amplicon sequencing (PCR with universal primer pairs Bact_541F/805R and A519F/U906R); Illumina MiSeq, 2x250 bp  RNA: Illumina Miseq, 2x100 bp |
| **Assembly Method** | Metatranscriptome Assembly using MEGAHIT |
| **16S rRNA gene analysis** | CLC Genomic Workbench software 10.0.1 with the additional microbial genomic module 2.0 |
